# Unmet need for contraception and its associated factors among women in Papua New Guinea: analysis from the demographic and health survey

**DOI:** 10.1186/s12978-022-01417-7

**Published:** 2022-05-08

**Authors:** Amma Kyewaa Agyekum, Kenneth Setorwu Adde, Richard Gyan Aboagye, Tarif Salihu, Abdul-Aziz Seidu, Bright Opoku Ahinkorah

**Affiliations:** 1 Department of Construction Technology and Management, Kwame Nkrumah University of Science and Technology, Kumasi, Ghana; 2 Department of Population and Health, University of Cape Coast, Cape Coast, Ghana; 3 Department of Family and Community Health, School of Public Health, University of Health and Allied Sciences, Hohoe, Ghana; 4 College of Public Health, Medical and Veterinary Sciences, James Cook University, Townsville, Australia; 5 Department of Real Estate Management, Takoradi Technical University, P.O.Box 256, Takoradi, Ghana; 6 Centre for Gender and Advocacy, Takoradi Technical University, P.O. Box 256, Takoradi, Ghana; 7 School of Public Health, Faculty of Health, University of Technology Sydney, Sydney, Australia

**Keywords:** Unmet need, Contraception, Prevalence, Maternal health, Papua New Guinea

## Abstract

**Background:**

Unmet need for contraception is highest in low-and middle-income countries. In Papua New Guinea, about 26% of married women and 65% of unmarried sexually active women have an unmet need for contraception. This study investigated the prevalence and correlates of unmet need for contraception among women in Papua New Guinea.

**Methods:**

Data for the study were extracted from the most recent 2016–18 Papua New Guinea Demographic and Health Survey. We included 7950 women with complete data on all variables of interest. Multilevel logistic regression analysis was conducted to examine the factors associated with unmet needs for contraception using four models. Adjusted odds ratios (AOR) and 95% confidence intervals (CIs) were used to present the  results of the regression analysis.

**Results:**

We found that the overall unmet need for contraception was 32.2%. The odds of unmet needs for contraception was higher among cohabiting women [AOR = 1.25, 95% CI = 1.01, 1.56], women with 1–3 births [AOR = 1.57, 95% CI = 1.18, 2.08], and women with 4 or more births [AOR = 1.06, 95% CI = 1.13, 2.27]. Likewise, a higher probability of unmet need was found among women whose partners decided on their healthcare as compared to those who decided on their own healthcare [AOR = 1.35, 95% CI = 1.066, 1.71]. With regards to wealth, the likelihood of unmet contraceptive need decreased with an increase in wealth status. With region, it was found that women in the Mamose region had greater likelihood of unmet contraceptive need compared to those in Southern region [AOR = 1.33, 95% CI = 1.09, 1.63].

**Conclusion:**

Our study contributes to the discussion on unmet need for contraception in the context of Papua New Guinea. We found the overall prevalence of unmet need for contraception to be relatively high among women in Papua New Guinea. Public health interventions aimed at addressing women's contraception needs should be encouraged so that women can make informed decisions about contraceptive use. These interventions should be implemented taking into consideration significant socio-demographic characteristics of women as identified in this study.

## Plain language summary

Unmet need for contraception is highest in low-and middle-income countries. This study investigates the prevalence and correlates of unmet need for contraception among women in Papua New Guinea. We extracted data from the most recent Demographic and Health Survey conducted in Papua New Guinea. Our study involved 7950 women with diverse contraceptive needs and those who have complete data on all variables of interest. We found that the overall unmet need for contraception was 32.2%. Marital status, parity, decision maker on respondent’s healthcare, wealth status, and region were found to have a significant relationship with unmet need for contraception. Cohabiting women recorded a higher likelihood for unmet need for contraception as compared to those married. Women with at least on child showed greater probability of unmet contraceptive need relative to women without children. Likewise, a higher probability of unmet need was found among women whose partners decided on their healthcare as compared to those who decided on their own healthcare. With regards to wealth, the likelihood of unmet contraceptive need decreased with an increase in wealth status. With region, it was found that women in the Mamose region had greater likelihood of unmet contraceptive need compared to those in Southern region. Interventions aimed at reducing unmet need for contraception should be implemented taking into consideration significant socio-demographic characteristics of women as identified in this study.

## Introduction

The use of contraceptives as a relevant fertility regulator has been accepted globally and serves as a significant factor in the health and wellbeing of women [1]. Furthermore, maternal health as a topical issue is highlighted under the Sustainable Development Goal (SDG) 3 which focuses on ensuring global access to sexual and reproductive health services and reducing maternal mortality by 2030 [2, 3]. These unmet needs contribute to unsafe abortions, sexually transmitted infections, increased fertility rate connected to poverty, high maternal mortality rates, and low employment [4].

Unmet need for contraception is defined by Bradley [4] as the percentage of sexually active women (married or cohabiting) or fecund women not using any form of contraception but wish to postpone their next birth for a minimum of 2 years or halt childbearing permanently. Kantorová et al. [5] revealed that in 2019, women of reproductive age group (15–49 years) were 1.9 billion, 1.1 billion of them had a need for family planning; out of these, 270 million had an unmet need for contraception while about 800 million relied on contraception. Projections made revealed the worldwide unmet need for family planning to be above 10% by 2030 [6]. The pronounced cause of unmet need is globally attributed to inadequate knowledge on contraceptive usage, side effects of contraceptives, health concerns, behavioural requirements and objections from husbands, religious or cultural restraints [7, 8]. Generally, unmet need for contraception or family planning is recorded to be higher in women living in rural areas, young women and those with low level of education [9].

Wemakor et al. [9] opined that unmet need for contraception is common in low and middle-income countries with scanty information on its determinants. Papua New Guinea (PNG) is an island country to the North of Australia with a low middle income. PNG is the largest Pacific Island country in terms of land area and the only Pacific Island country that shares a border with Indonesia. It is one of the countries with sub optimal health outcomes [10]. Evidence suggests that the incidence of unmet need for contraception among women in unions between 15 and 49 years did not improve in PNG during the two decades from 1990 to 2010, making it one of 42 nations worldwide with an unmet need of more than 25% [6]. However, it has reduced a bit from 37% in 2006 to 32% in 2016–2018 [11]. Specifically, it is estimated that as of 2015, about 317,000 women of reproductive age in PNG had unmet need for contraception and this number is expected to rise to 337,000 by 2030 [12]. The 2016–2018 Demographic and Health Survey (DHS) in PNG (PNG DHS) indicated that about 26% of married women and 65% of unmarried sexually active women have an unmet need for contraception [13]. Unmet need for contraception could prevent nearly half (47.4%) of all maternal fatalities in PNG [14]. Although, maternal mortality has reduced over the years in PNG, with 733 deaths per 100, 000 births in 2006 to 171/100,000 deaths per birth currently, this is still high and urgent attention in terms of policies and improving maternal health [11, 12].

There is therefore, more room for improvement in attaining the SDG of ensuring the health and wellbeing of all [11, 13]. There is limited research on unmet need for contraception among women in PNG. PNG has a record of inadequate basic health care, shortage of medical supplies, lack of skilled health personnel to provide required services and limited policies to facilitate maternal health [15]. However, attaining adequate access to contraception is a key mandate of the PNG National Health Plan in achieving the SDGs and reducing maternal mortality rate by 2030. In an attempt to recommend measures to policy creators, this study seeks to investigate the prevalence and correlates of unmet need for contraception among women in PNG.

## Methods

### Data source and study design

This study involved a secondary analysis of the 2016–18 PNG DHS which was collected using a cross-sectional study design. PNG DHS collects data from respondents on health and social indicators including contraceptive use. The data was collected using a structured questionnaire. PNG DHS employed a two-stage cluster sampling technique to recruit the respondents for the survey. The first stage entailed the selection of 800 census units (CUs). This was accomplished by using a probability proportional to the size of the CU. The second stage involved selecting 24 households from each cluster using random sampling, resulting in a total of 19,200 households. The detailed study methodology can be found at https://dhsprogram.com/publications/publication-fr364-dhs-final-reports.cfm. In this study, a total of 7950 women of reproductive age (15-49) with complete cases on variables of interest were included in the study. The PNG DHS dataset can be freely accessed at https://dhsprogram.com/data/dataset_admin/index.cfm.

## Variables

### Outcome variable

Unmet need for contraception was the outcome variable in this study. From the DHS, unmet need was the aggregated sum of unmet needs for spacing and limiting and reproductive-age women who were married, fecund, and/or sexually active have unmet needs if they do not want any more children or want to postpone their next birth for at least 2 years but are not using contraception. Also, pregnant or amenorrheic women who had unwanted or mistimed pregnancies or births were considered to have unmet need if they did not use contraception at the time they conceived [16–19]. Unmet need was coded as a binary variable; thus, no = 0 and yes = 1 and used in the final analysis. This coding was based on studies that used the DHS dataset [16–18].

### Explanatory variables

Explanatory variables included in the study were selected based on their significant association with unmet needs for contraception [16–19] and their availability in the DHS dataset. The variables were grouped into the individual level and household/community level (contextual) variables based on the review of literature that utilized the DHS dataset [16, 18]. The individual level variables consisted of age of the women (15–19, 20–24, 25–29, 30–34, 35–39, 40–44, 45–49), educational level (no education, primary, secondary, higher), marital status (married, cohabiting), current working status (yes, no), parity (0, 1–3, 4 or more), frequency of watching television (not at all, less than once a week, at least once a week), frequency of listening to radio (not at all, less than once a week, at least once a week), frequency of reading newspaper or magazine (not at all, less than once a week, at least once a week), heard of family planning from newspaper or magazine in the last few months (yes, no), heard of family planning from radio in the last few months (yes, no), heard of family planning from television in the last few months (yes, no), and person who usually decides on respondent’s health care (respondent alone, respondent and partner, partner alone, someone else or other). DHS calculated the wealth index based on the ownership of family assets, such as access to drinking water, kind of toilet, type of cooking fuel, and possession of a television and refrigerator. A principal component analysis was adopted for the creation of the wealth index. Based on this, wealth index was divided into five categories based on individual rankings: poorest, poorer, middle, richer, and richest. Sex of household head (male, female), place of residence (urban, rural), and region (Southern, Highlands, Momase, Islands) were the household/ community level variables used in the study.

### Statistical analyses

Data extraction, cleaning, and analysis were carried out using Stata software version 16.0. Percentages were used to summarise the prevalence of unmet needs for contraception among the respondents. Cross-tabulation was later adopted to examine the distribution of unmet needs across the explanatory variables. The results of the cross-tabulation were presented using percentages with their corresponding confidence intervals. Also, Pearson chi-square test was used to examine the explanatory variables significantly associated with unmet needs for contraception. Next, we carried out a multicollinearity test and realized no collinearity among the explanatory variables (minimum = 1.04, mean = 2.40, maximum = 5.41). Subsequently, a multivariable multilevel regression analysis was performed to determine the factors associated with unmet needs for contraception. Four models were built to examine the factors associated with unmet needs. The first model (Model O) consisted of only unmet needs (outcome variable). The results signified the variance in unmet needs attributed to the clustering at the primary sampling units (PSUs). Model I was built to contain the individual-level variables whilst Model II consisted of the household/community level variables. In Model III, all the explanatory variables were put together to examine their association with unmet needs. The results were presented using adjusted odds ratio (AOR) with their respective 95% confidence intervals. We used Akaike's Information Criterion (AIC) to analyze model fitness and model comparability.The women's sample weights (v005/1,000,000) were utilized to achieve unbiased estimates. The Stata survey command 'svy' was used to correct for the data's complex sampling structure in the chi-square and regression analyses as recommended by the DHS.

### Ethical consideration

Ethical approval was not sought for this study since publicly available data was used. However, the 2016–18 PNG DHS stated that the ICF Institutional Review Board granted ethical clearance. Written informed consent was obtained during data collection. In this study, we complied with the guidelines concerning the use of secondary data for publication. Further information about the data and ethical standards can be assessed at http://goo.gl/ny8T6X.

## Results

### Socio-demographic characteristics and prevalence of unmet need for contraception

Table 1 provides the results of the distribution of unmet need for contraception among women in PNG by the explanatory variables. The overall prevalence of unmet needs for contraception was 32.2%.   A high prevalence of unmet need for contraception (38.0%) was found among women with no education. Unmet contraceptive need was prevalent among cohabiting women (37.9%). In terms of mass media, women who did not watch television at all (34.3%), those who did not listen to radio at all (35.5%), and those who failed to read newspaper or magazine at all (34.9%) had higher proportion of unmet contraceptive need. It was also revealed that women who did not hear of family planning from newspaper or magazine in the last few months (33.1%), those who did not hear of family planning from radio in the last few months (33.2%), and those who did not hear of planning from television in the last few months (32.8%) recorded higher prevalence of unmet contraceptive need. High proportion of unmet need was also found among persons who usually decided on respondent partner’s healthcare alone (39.0%). With wealth index, the highest prevalence of unmet contraceptive need was observed among women in the poorest wealth quintile (41.3%), whereas the lowest was observed among those in the richest wealth quintile (24.4%). High prevalence of unmet need was also found among rural women (33.2%). In terms of place of residence, women in the Mamose region recorded the highest proportion of unmet contraceptive need (35.5%) whereas women in the Southern region recorded the lowest (28.3%). Finally, all the explanatory variables considered in the analysis were significantly related with unmet contraceptive need with the exception of age, current working status, parity, and sex of household head (see Table 1).

**Table 1:** Distribution of unmet need across explanatory variables

Variable	Weighted N	Weighted %	Unmet needs for contraception		
			No (%) 67.8%	Yes (%) 32.2%	P-value
*Women’s age (years)*					0.096
15–19	365	4.6	65.2 [57.6, 72.2]	34.8 [27.8, 42.4]	
20–24	1472	18.5	64.0 [59.9,68.0]	36.0 [32.0, 40.1]	
25–29	1820	22.9	69.8 [66.6, 72.8]	30.2 [27.2, 33.4]	
30–34	1554	19.5	70.2 [67.0, 73.2]	29.8 [26.8, 33.0]	
35–39	1371	17.3	68.1 [64.0, 72.0]	31.9 [28.0, 36.0]	
40–44	854	10.7	64.7 [60.2, 69.0]	35.3 [31.0, 39.8]	
45–49	514	6.5	70.5[65.1, 75.4]	29.5 [24.6, 34.9]	
*Level of education*					0.003
No education	1982	24.9	62.0 [57.9, 65.9]	38.0 [34.1, 42.1]	
Primary	3928	49.4	67.6 [65.7, 69.5]	32.4 [30.5, 34.3]	
Secondary	1716	21.6	74.3 [71.3, 77.1]	25.7 [22.9, 28.7]	
Higher	324	4.1	71.6 [57.0, 82.7]	28.4 [17.3, 43.0]	
*Marital status*					0.004
Married	6594	82.9	69.0 [67.2, 70.7]	31.0 [29.3, 32.8]	
Cohabiting	1356	17.1	62.1 [57.6, 66.4]	37.9 [33.6, 42.4]	
*Current working status*					0.914
No	5389	67.8	67.9 [66.0, 69.7]	32.1 [30.3, 34.0]	
Yes	2561	32.2	67.7 [64.8, 70.5]	32.3 [29.5,35.2]	
*Parity*					0.077
Zero	632	8.0	74.3 [68.5,79.3]	25.7 [20.7,31.5]	
1–3 births	4119	51.8	67.6 [65.4,69.7]	32.4 [30.3,34.6]	
4 or more births	3199	40.2	66.8 [64.0,69.6]	33.2 [30.4,36.0]	
*Frequency of watching television*					< 0.001
Not at all	6139	77.2	65.7 [63.7,67.6]	34.3 [32.4,36.3]	
Less than once a week	729	9.2	74.7 [68.8,79.8]	25.3 [20.2,31.2]	
At least once a week	1082	13.6	75.3 [71.6,78.7]	24.7 [21.3,28.4]	
*Frequency of listening to radio*					< 0.001
Not at all	5118	64.4	64.5 [62.3,66.7]	35.5 [33.3,37.7]	
Less than once a week	1506	18.9	72.6 [69.4,75.6]	27.4 [24.4,30.6]	
At least once a week	1326	16.7	75.1 [71.7,78.2]	24.9 [21.8,28.3]	
*Frequency of reading newspaper or magazine*					
Not at all	5198	65.4	65.1 [63.0,67.2]	34.9 [32.8,37.0]	
Less than once a week	1467	18.4	69.7 [65.5,73.6]	30.3 [26.4,34.5]	
At least once a week	1285	16.2	76.7 [72.7,80.3]	23.3 [19.7,27.3]	
*Heard of family planning from newspaper or magazine last few months*					0.009
No	6975	87.7	66.9 [65.1,68.7]	33.1 [31.3,34.9]	
Yes	975	12.3	74.1 [69.2,78.5]	25.9 [21.5,30.8]	
*Heard of family planning from radio last few months*					< 0.001
No	6912	86.9	66.8 [65.0,68.6]	33.2 [31.4,35.0]	
Yes	1038	13.1	74.5 [71.2,77.6]	25.5 [22.4,28.8]	
*Heard of family planning from television last few months*					0.002
No	7314	92.0	67.2 [65.4,68.9]	32.8 [31.1,34.6]	
Yes	636	8.0	75.3 [70.8,79.4]	24.7 [20.6,29.2]	
*Person who usually decides on respondent’s health care*					0.031
Respondent alone	2346	29.5	67.3 [63.8,70.6]	32.7 [29.4,36.2]	
Respondent and partner	4392	55.2	70.0 [67.6,72.2]	30.0 [27.8,32.4]	
Partner alone	1080	13.6	61.0 [55.9,65.8]	39.0[34.2,44.1]	
Someone else or other	132	1.7	63.0 [43.9,78.7]	37.0 [21.3,56.1]	
*Wealthindex*					< 0.001
Poorest	1471	18.5	58.7 [55.0,62.3]	41.3 [37.7,45.0]	
Poorer	1529	19.2	64.0 [60.1,67.7]	36.0 [32.3,39.9]	
Middle	1582	19.9	68.2 [64.5,71.7]	31.8 [28.3,35.5]	
Richer	1656	20.8	71.0 [67.9,73.9]	29.0 [26.1,32.1]	
Richest	1712	21.5	75.6 [73.0,78.1]	24.4 [21.9,27.0]	
*Sex of household head*					0.224
Male	6916	87.0	67.4 [65.7,69.1]	32.6 [30.9,34.3]	
Female	1034	13.0	70.4 [65.6,74.8]	29.6 [25.2,34.4]	
*Place of residence*					< 0.001
Urban	1011	12.7	75.0 [72.3,77.5]	25.0 [22.5,27.7]	
Rural	6939	87.3	66.8 [64.9,68.6]	33.2 [31.4,35.1]	
*Region*					0.009
Southern region	1583	19.9	71.7 [69.1,74.1]	28.3 [25.9,30.9]	
Highlands region	3007	37.8	67.0 [63.8,70.1]	33.0 [29.9,36.2]	
Momase region	2172	27.3	64.5 [61.3,67.6]	35.5 [32.4,38.7]	
Islands region	1188	14.9	70.7 [66.9,74.1]	29.3 [25.9,33.1]	

Model III of Table 2 summarizes the multilevel analysis of factors associated with unmet need for contraception among women in PNG. The analysis reveals higher likelihood of unmet contraceptive need among cohabiting women compared to those married [AOR = 1.25, 95% CI = 1.01, 1.56]. In terms of parity, women with 1–3 births [AOR = 1.57, 95% CI = 1.18, 2.08] and women with 4 or more births [AOR = 1.60, 95% CI = 1.13, 2.27] showed greater probability of unmet contraceptive need relative to women without births. A higher probability of unmet need was found among women whose partners decided on their healthcare as compared to those who decided on their own healthcare [AOR = 1.35, 95% CI = 1.06, 1.71]. The likelihood of unmet contraceptive need decreased with an increase in wealth status. Particularly, women in the richest wealth quintile had the lowest unmet contraceptive need relative to those in the poorest wealth quintile [AOR = 0.59, 95% CI = 0.43, 0.80]. With region, it was discovered that women in the Mamose region had greater likelihood of unmet contraceptive need compared to those in the Southern region [AOR = 1.33, 95% CI = 1.09, 1.63].

**Table 2:** Fixed and random effect analysis of factors associated with unmet needs for contraception

Variable	Model O	Model I AOR [95% CI]	Model II AOR [95% CI]	Model III AOR [95% CI]
**Fixed effect results**				
*Women’s age (years)*				
15–19		1 [1.00,1.00]		1 [1.00,1.00]
20–24		1.02 [0.69,1.52]		1.04 [0.70,1.55]
25–29		0.69 [0.45,1.06]		0.70 [0.46,1.08]
30–34		0.68 [0.44,1.06]		0.70 [0.45,1.09]
35–39		0.70 [0.44,1.12]		0.73 [0.45,1.17]
40–44		0.82 [0.53,1.29]		0.87 [0.55,1.37]
45–49		0.64 [0.39,1.03]		0.67 [0.41,1.08]
*Level of education*				
No education		1 [1.00,1.00]		1 [1.00,1.00]
Primary		0.92 [0.76,1.12]		0.97 [0.80,1.18]
Secondary		0.78 [0.61,1.00]		0.87 [0.67,1.13]
Higher		1.09 [0.48,2.45]		1.27 [0.56,2.88]
*Marital status*				
Married		1 [1.00,1.00]		1 [1.00,1.00]
Cohabiting		1.28^*^ [1.03,1.60]		1.25^*^ [1.01,1.56]
*Current working status*				
No		1 [1.00,1.00]		1 [1.00,1.00]
Yes		1.08 0.93,1.26]		1.09 [0.94,1.28]
*Parity*				
Zero		1 [1.00,1.00]		1 [1.00,1.00]
1–3 births		1.57^**^ [1.19,2.09]		1.57^**^ [1.18,2.08]
4 or more births		1.64^**^ [1.15,2.33]		1.60^**^ [1.13,2.27]
*Frequency of watching television*				
Not at all		1 [1.00,1.00]		1 [1.00,1.00]
Less than once a week		0.80 [0.56,1.14]		0.86 [0.60,1.23]
At least once a week		0.90 [0.66,1.23]		1.01 [0.73,1.39]
*Frequency of listening to radio*				
Not at all		1 [1.00,1.00]		1 [1.00,1.00]
Less than once a week		0.79 [0.61,1.02]		0.80 [0.61,1.04]
At least once a week		0.78 [0.56,1.07]		0.80 [0.59,1.10]
*Frequency of reading newspaper or magazine*				
Not at all		1 [1.00,1.00]		1 [1.00,1.00]
Less than once a week		1.11 [0.85,1.44]		1.13 [0.86,1.49]
At least once a week		0.77 [0.53,1.12]		0.81 [0.56,1.17]
*Heard of family planning from newspaper or magazine last few months*				
No		1 [1.00,1.00]		1 [1.00,1.00]
Yes		1.02 [0.69,1.50]		1.05 [0.71,1.54]
*Heard of family planning from television last few months*				
No		1 [1.00,1.00]		1 [1.00,1.00]
Yes		1.02 [0.61,1.71]		1.05 [0.63,1.75]
*Heard of family planning from radio last few months*				
No		1 [1.00,1.00]		1 [1.00,1.00]
Yes		0.93 [0.72,1.21]		0.94 [0.73,1.22]
*Person who usually decides on respondent’s health care*				
Respondent alone		1 [1.00,1.00]		1 [1.00,1.00]
Respondent and partner		0.87 [0.72,1.06]		0.86 [0.71,1.04]
Partner alone		1.37^*^ [1.08,1.74]		1.35^*^ [1.06,1.71]
Someone else or other		1.20 [0.55,2.61]		1.19 [0.55,2.57]
*Wealthindex*				
Poorest			1 [1.00,1.00]	1 [1.00,1.00]
Poorer			0.85 [0.67,1.08]	0.86 [0.68,1.10]
Middle			0.73^**^ [0.58,0.91]	0.75^*^ [0.60,0.94]
Richer			0.65^***^ [0.51,0.83]	0.69^**^ [0.53,0.90]
Richest			0.51^***^ [0.40,0.66]	0.59^***^ [0.43,0.80]
*Sex of household head*				
Male			1 [1.00,1.00]	1 [1.00,1.00]
Female			0.86 [0.68,1.09]	0.87 [0.68,1.10]
*Place of residence*				
Urban			1 [1.00,1.00]	1 [1.00,1.00]
Rural			1.15 [0.94,1.42]	1.08 [0.87,1.33]
*Region*				
Southern region			1 [1.00,1.00]	1 [1.00,1.00]
Highlands region			1.07 [0.89,1.29]	1.05 [0.86,1.27]
Momase region			1.35^**^ [1.11,1.64]	1.33^**^ [1.09,1.63]
Islands region			1.08 [0.88,1.33]	1.10 [0.89,1.36]
*Random effect model*				
PSU variance (95% CI)	0.398 [0.299, 0.531]	0.343 [0.251, 0.471]	0.328 [0.237, 0.453]	0.322 [0.232, 0.447]
ICC	0.1079641	0.0945348	0.0905374	.0891375
Wald chi-square	Reference	115.40 (< 0.001)	86.56 (< 0.001)	169.18 (< 0.001)
*Model fitness*				
Log-likelihood	− 4839.6637	− 4755.7949	− 4799.8545	− 4735.382
AIC	9683.327	9565.59	9621.709	9542.764
N	7950	7950	7950	7950
Number of clusters	763	763	763	763

Exponentiated coefficients; 95% confidence intervals in brackets; *AOR* adjusted odds ratios, *CI* confidence interval; 1 = Reference category; *PSU* primary sampling unit; *ICC*  intra-class correlation, *AIC*  Akaike’s Information Criterion. ^*^p < 0.05, ^**^p < 0.01, ^***^p < 0.001

## Discussion

The present study sought to examine the prevalence and factors associated with unmet need for contraception among women in PNG. We found that the overall unmet need for contraception was 32.2%. The prevalence observed in the present study is consistent with prior research in Burundi [20], Cameroon [21], Ghana [22] and PNG [10]. However, the result is higher than what was found in several previous studies including 11.5% in Mexico [23], 16.2% in Ethiopia [24], 18% in Northern Nigeria [25], 12.7% in Egypt [26] and 17% in Indonesia [27].The significant prevalence of unmet contraceptive needs in the current study relative to earlier studies could be attributed to the varied target group and sample size in this and prior studies as well as socio-cultural norms and gender inequity which prevent women in PNG from accessing contraceptives [10, 28].

The study revealed that cohabiting women exhibited a higher likelihood of unmet contraceptive needs compared to married women. This finding corroborates prior studies in Nigeria [29], Mexico [23] and sub-Saharan Africa [17] where cohabiting women were more likely to have unmet contraceptive need than married women. One possible reason for higher odds among cohabiting women could be due to objection from partners [26, 30]. Additionally, it could be that in most countries including PNG, the need for childbearing is restricted during cohabitation relative to wedlock because of socio-cultural values about childbearing outside of marriage [31, 32]. However, this finding contrast those of Klinzing [33] in Hungary, who reported that married women had a greater likelihood of unmet contraceptive need.

In this study, high unmet contraceptive need was realized among women with 1–3 children and those with four or more children compared with women without children. Thus, the study found a positive association between parity and unmet need for contraception. This result is in line with prior studies conducted in low-and middle-income countries [34], sub-Saharan Africa [17], Nigeria [25], and Pakistan [7] which indicated that as the number of kids for a woman increase, so does her unmet contraceptive need. This could be attributed to strong patriarchal mores, a higher priority placed on male children than female children, and mostly rural systems assuring economic or social stability centered on huge family sizes that demand women to bear numerous children [35, 36]. Prior studies conducted in Burkina Faso [37, 38], Egypt [26], Ethiopia [18], Zambia [39], and India [40], however, showed a negative relationship between parity and unmet need for contraception. The reason for this could be that as the overall number of children upsurges, peoples’ craving to have more children decreases [41].

The present study found women whose partners usually decided on their healthcare to be significantly associated with unmet need for contraception. That is, women whose partners had an ultimate decision on their healthcare showed a greater probability of unmet contraceptive need compared to those who decided on their own healthcare. This finding is comparable with other studies in Burundi [20] and Ghana [42] which revealed that partners decisions towards their wives’ reproductive health such as denial of contraceptive use due to desire for children augmented the likelihood of unmet contraceptive need for them. Partners are frequently wary of approving any contraceptive use for fear of losing their position as family heads and/or implicitly pushing their women to be disloyal or promiscuous [36]. In sub-Saharan Africa, men are usually the ones who make decisions about their wives’ healthcare due to sociocultural norms and a lack of economic independence among women [21]. Males should, therefore, be included in all phases of the sexual and reproductive health program (SRHP) so that they may be educated to back their women in utilizing contraceptives and foster inter-spousal fertility discourse [42]. This finding is however, contrary to other Ethiopian studies [24, 43], which found that women who made decisions on their own health with practitioners showed a reduced likelihood of unmet contraceptive need. It is worth noting that different women have various health concerns and reproductive health goals, so making contraceptive decision on their own was preferable as making an informed decision was good for continued use of the contraceptive method of their choice [24].

The current study also found wealth status to be inversely associated with an unmet need for contraception. Women in the richest wealth quintile were discovered to have the lowest likelihood of unmet contraceptive need compared to their counterparts in the poorest wealth quintile. This indicates that the odds of unmet need reduced with augmented in wealth status. A similar relationship was found in prior studies conducted in different parts of the globe including Libya [44], Nigeria [45], Pakistan [7], Ethiopia [24], PNG [10] and sub-Saharan Africa [16]. One possible reason is that women from wealthy families have more accessible contact with contemporary contraceptives than those from poorer families because they could afford both the direct and indirect costs of contraceptive use [7].

Furthermore, women in this group are more likely to be informed about contraceptive use [15]. However, the results of this research are inconsistent with other studies in Ethiopia [24, 46] where they discovered that affluent women had higher odds of unmet contraceptive needs. This indicates that having a high wealth index does not assure that your contraception needs will be addressed.

The current study discovered that geographic location has a statistically significant relationship with unmet contraceptive needs. This finding showed that there were variations among various regions of PNG for unmet contraceptive need. The Mamose region recorded the highest rate of unmet contraceptive needs, indicating healthcare gaps in the delivery of critical family planning services in one of the country's most populous areas. Compared with women staying in the Southern region, those in the Mamose region were found to have greater risk of unmet contraceptive need. This could be due to geographic barricades and a broadly dispersed population, which impedes the smooth delivery of reproductive health services [10] in the region where women have habitually had the maximum fertility levels, but now have a bigger need to use contraception to delay childbearing [23]. It may also be challenging to deliver contraceptive services to address the unmet contraceptive need due to sensitivities regarding sexual and reproductive health, cultural obstacles, and religious beliefs [10]. It is worth mentioning that unmet contraceptive needs vary by location due to societal differences and differences in geographic and economic access to reproductive health services for women [46, 47].

### Strengths and limitations

One of the study's strengths is the utilization of a nationally representative dataset to examine the factors associated with  unmet contraception needs among women in PNG. Also, in cross sectional studies, the use of large sample size is relatively important and this has helped to strengthen the validity and generalizability of the study findings. Furthermore, the results obtained using scientific study standards and appropriate techniques perfectly agree with past research findings. This study would help PNG allocate contraceptive resources more effectively by indicating contraceptive needs. However, women may give socially acceptable responses and may find it difficult to recall past experiences, resulting in recall bias in the study.

### Policy implications

The significant level of unmet need for contraception necessitates immediate program retorts and intercessions from the health division to improve contraceptive availability and use for every woman in PNG. According to previous studies, well-organized contraceptive service programs reduce unmet contraceptive needs directly and indirectly [48, 49]. The PNG government should strengthen inclusion of family planning services and contraception delivery as part of universal health coverage (UHC), focusing on the very susceptible groups, including rural women. Beyond the health sector, action must be taken to promote societal norms and policy measures that promote sexual and reproductive liberties. Collaboration and partnership between public health services and faith-based health services should be fostered even more in order to improve access to a variety of contemporary contraceptive techniques at a low cost [10]. The findings of this study could help the PNG government track progress toward UHC at the national and regional stages. Currently, the WHO [50] indicates that PNG UHC index on family planning and reproductive health in general is very low. In addition, from the WHO SDG performance score care for the Asia Pacific region, PNG has attained only 7% as far as the need for family planning is concerned. This finding is also a wakeup call to the government to step up their game on reproductive health services provision in PNG [51, 52].

## Conclusions

The overall prevalence of unmet need for contraception was found to be high in this study. This emphasizes the necessity for the country’s contraception programs to be redesigned. This study also found some critical factors associated with unmet contraceptive needs among Papua New Guinean women. Marital status, parity, the person who normally decides on a respondent’s healthcare, wealth status, and region all showed significant association with unmet contraceptive need. We believe that public health interventions aimed at addressing women’s contraception needs should be encouraged to make informed decisions about contraception use. Contraceptive services should be prioritized for cohabiting women, women in the poorest wealth quintile, women with multiple children, and women in the country’s poorest regions. Further studies could explore the association between women's autonomy, gender-based violence and their association with contraception use in PNG.

## Data Availability

https://dhsprogram.com/data/dataset_admin/index.cfm.

## Abbreviations

AOR: Adjusted odds ratio
AIC: Akaike's information criterion
CI: Confidence interval
PSU: Primary sampling unit
ICC: Intra-class correlation
SDG: Sustainable development goal
DHS: Demographic and Health Survey
PNG: Papua New Guinea
PNGDHS: Papua New Guinea Demographic and Health Survey

## References

[1] Ahmed S, Li Q, Liu L, Tsui AO (2012). Maternal deaths averted by contraceptive use: an analysis of 172 countries. Lancet.

[2] World Health Organization. Health in 2015: from MDGs, millennium development goals to SDGs, sustainable development goals.

[3] Grove J, Claeson M, Bryce J, Amouzou A, Boerma T, Waiswa P, Victora C (2015). Maternal, newborn, and child health and the Sustainable Development Goals—a call for sustained and improved measurement. The Lancet.

[4] Bradley SE. Revising unmet need for family planning. ICF International; 2012.

[5] Kantorová V, Wheldon MC, Ueffing P, Dasgupta AN (2020). Estimating progress towards meeting women’s contraceptive needs in 185 countries: a Bayesian hierarchical modelling study. PLoS Med.

[6] Alkema L, Kantorova V, Menozzi C, Biddlecom A (2013). National, regional, and global rates and trends in contraceptive prevalence and unmet need for family planning between 1990 and 2015: a systematic and comprehensive analysis. Lancet.

[7] Asif MF, Pervaiz Z (2019). Socio-demographic determinants of unmet need for family planning among married women in Pakistan. BMC Public Health.

[8] Krakowiak-Redd D, Ansong D, Otupiri E, Tran S, Klanderud D, Boakye I, Dickerson T, Crookston B (2011). Family planning in a sub-district near Kumasi, Ghana: side effect fears, unintended pregnancies and misuse of a medication as emergency contraception. Afr J Reprod Health.

[9] Wemakor A, Garti H, Saeed N, Asumadu O, Anyoka B (2020). Prevalence and determinants of unmet need for contraception in North Gonja district, Ghana. BMC Womens Health.

[10] Pham BN, Whittaker M, Okely AD, Pomat W (2020). Measuring unmet need for contraception among women in rural areas of Papua New Guinea. Sex Reprod Health Matters.

[11] Seidu AA, Agbaglo E, Dadzie LK, Ahinkorah BO, Ameyaw EK, Tetteh JK, Yaya S (2020). Modern contraceptive utilization and associated factors among married and cohabiting women in Papua New Guinea: a population-based cross-sectional study. Contracept Reprod Med.

[12] United Nations, Department of Economic and Social Affairs, Population Division. 2015. Trends in Contraceptive Use Worldwide 2015 (ST/ESA/SER.A/349).

[13] Thanenthiran S. Access to contraception and family planning in Asia and the Pacific region. Asian-Pacific resource and research center for women. 2019.

[14] Ahmed S, Choi Y, Rimon JG, Alzouma S, Gichangi P, Guiella G (2019). Trends in contraceptive prevalence rates in sub-Saharan Africa since the 2012 London Summit on Family Planning: results from repeated cross-sectional surveys. Lancet Glob Health.

[15] Gupta S, McGeechan K, Bernays S, Mola G, Kelly-Hanku A, Bolnga JW, Black KI (2021). Fertility preferences, contraceptive use, and the unmet need for contraception in Papua New Guinea: key findings from 1996 to 2016. Asia Pac J Public Health.

[16] Ahinkorah BO (2020). Predictors of unmet need for contraception among adolescent girls and young women in selected high fertility countries in sub-Saharan Africa: a multilevel mixed effects analysis. PLoS ONE.

[17] Ahinkorah BO, Ameyaw EK, Seidu AA (2020). Socio-economic and demographic predictors of unmet need for contraception among young women in sub-Saharan Africa: evidence from cross-sectional surveys. Reprod Health.

[18] Yalew M, Adane B, Kefale B, Damtie Y (2020). Individual and community-level factors associated with unmet need for contraception among reproductive-age women in Ethiopia; a multi-level analysis of 2016 Ethiopia Demographic and Health Survey. BMC Public Health.

[19] Dingeta T, LemessaOljira AW, Berhane Y (2019). Unmet need for contraception among young married women in eastern Ethiopia. Open Access J Contracept.

[20] Nzokirishaka A, Itua I (2018). Determinants of unmet need for family planning among married women of reproductive age in Burundi: a cross-sectional study. Contracept Reprod Med.

[21] Edietah EE, Njotang PN, Ajong AB, Essi MJ, Yakum MN, Mbu ER (2018). Contraceptive use and determinants of unmet need for family planning; a cross sectional survey in the North West Region, Cameroon. BMC Womens Health.

[22] Wulifan JK, Mazalale J, Kambala C, Angko W, Asante J, Kpinpuo S, Kalolo A (2019). Prevalence and determinants of unmet need for family planning among married women in Ghana—a multinomial logistic regression analysis of the GDHS, 2014. Contracept Reprod Med.

[23] Juarez F, Gayet C, Mejia-Pailles G (2018). Factors associated with unmet need for contraception in Mexico: evidence from the National Survey of Demographic Dynamics 2014. BMC Public Health.

[24] Tadele A, Abebaw D, Ali R (2019). Predictors of unmet need for family planning among all women of reproductive age in Ethiopia. Contracept Reprod Med.

[25] Solanke BL, Oyinlola FF, Oyeleye OJ, Ilesanmi BB (2019). Maternal and community factors associated with unmet contraceptive need among childbearing women in Northern Nigeria. Contracept Reprod Med.

[26] Hassan EE, Ghazawy ER, Amein NM (2017). Currently married women with an unmet need for contraception in Minia governorate, Egypt: profile and determinants. Open J Prev Med.

[27] Ayuningtyas D, Oktaviana W (2015). Factors contributing to unmet need for contraception in Nusa Tenggara Barat, Indonesia. J Reprod Contracept.

[28] Bell MC, Edin K, Wood HM, Mondé GC (2018). Relationship repertoires, the price of parenthood, and the costs of contraception. Soc Service Rev.

[29] Oginni AB, Ahonsi BA, Adebajo S (2015). Trend and determinants of unmet need for family planning services among currently married women and sexually active unmarried women aged 15–49 in Nigeria (2003–2013). Afr Popul Stud.

[30] Khan HR, Shaw E (2011). Multilevel logistic regression analysis applied to binary contraceptive prevalence data. J Data Sci.

[31] Avogo WA, Somefun OD (2019). Early marriage, cohabitation, and childbearing in West Africa. J Environ Public Health.

[32] Odimegwu C, Frade S (2018). The influence of adolescent age at first union on physical intimate partner violence and fertility in Uganda: a path analysis. S Afr J Child Health.

[33] Klinzing ML (2012). Denying reparation for slave and forced laborers in World War II and the ensuing humanitarian rights implications: a case study of the ICJ's recent decision in jurisdictional immunities of the state (Ger. V. It.: Greece Intervening). Ga J Int Comp Law.

[34] Wulifan JK, Brenner S, Jahn A, De Allegri M (2015). A scoping review on determinants of unmet need for family planning among women of reproductive age in low and middle income countries. BMC Womens Health.

[35] Kabagenyi A, Jennings L, Reid A, Nalwadda G, Ntozi J, Atuyambe L (2014). Barriers to male involvement in contraceptive uptake and reproductive health services: a qualitative study of men and women’s perceptions in two rural districts in Uganda. Reprod Health.

[36] Mosha I, Ruben R, Kakoko D (2013). Family planning decisions, perceptions and gender dynamics among couples in Mwanza, Tanzania: a qualitative study. BMC Public Health.

[37] Adebowale SA, Palamuleni ME (2014). Determinants of unmet need for modern contraception and reasons for non-use among married women in rural areas of Burkina Faso. Afr Popul Stud.

[38] Wulifan JK, Jahn A, Hien H, Ilboudo PC, Meda N, Robyn PJ, Hamadou TS, Haidara O, De Allegri M (2017). Determinants of unmet need for family planning in rural Burkina Faso: a multilevel logistic regression analysis. BMC Pregnancy Childbirth.

[39] Imasiku EN, Odimegwu CO, Adedini SA, Ononokpono DN (2014). Variations in unmet need for contraception in Zambia: does ethnicity play a role?. J Biosoc Sci.

[40] Kandel NR (2012). Unmet need for contraception and its associated factors among married women of reproductive age in Simichaur VDC of Gulmi District. Health Prospect J Public Health.

[41] Hailemariam A, Haddis F (2011). Factors affecting unmet need for family planning in southern nations, nationalities and peoples region, Ethiopia. Ethiop J Health Sci.

[42] Staveteig S. Fear, opposition, ambivalence, and abstinence: understanding unmet need in Ghana. In 2016 Annual Meeting 2016 Apr 2. PAA.

[43] Chafo K, Doyore F. Unmet need for family planning and associated factors among currently married women in Misha District, southern Ethiopia: a cross sectional study. J Women’s Health Care. 2014;3(165):2167-0420

[44] Juárez F, Gayet C (2014). Transitions to adulthood in developing countries. Ann Rev Sociol.

[45] Fagbamigbe AF, Afolabi RF, Idemudia ES (2018). Demand and unmet needs of contraception among sexually active in-union women in Nigeria: distribution, associated characteristics, barriers, and program implications. SAGE Open.

[46] Dejenu G, Ayichiluhm M, Abajobir AA. Prevalence and associated factors of unmet need for family planning among married women in Enemay District, Northwest Ethiopia: a comparative cross-sectional study. Glob J Med Res. 2013;13(4).

[47] Mihret N. Magnitude and associated factors of unmet need for contraceptive methods among currently married women in west Belessa District, North Western Ethiopia. Int J Innov Res Dev. 2015;4(10).

[48] Cleland J, Harbison S, Shah IH (2014). Unmet need for contraception: issues and challenges. Stud Fam Plann.

[49] Bongaarts J (2014). The impact of family planning programs on unmet need and demand for contraception. Stud Fam Plann.

[50] World Health Organization. 2018. UHC and SDG country profiles 2018.

[51] Ahmed S, Li Q, Liu L, Tsui AO (2020). Maternal deaths averted by contraceptive use: an analysis of 172 countries. Lancet.

[52] Alkema L, Kantorova V, Menozzi C, Biddlecom A (2020). National, regional, and global rates and trends in contraceptive prevalence and unmet need for family planning between 1990 and 2015: a systematic and comprehensive analysis. Lancet.

